# Supplementary material for: Molecular basis of the pleiotropic effects by the antibiotic amikacin on the ribosome
Source: Nat Commun. 2023 Aug 3;14:4666. doi: 10.1038/s41467-023-40416-5 (PMC10400623; doi:10.1038/s41467-023-40416-5)
Supplement: Supplementary file 1 — Supplementary Information [file 41467_2023_40416_MOESM1_ESM.pdf]

Supplementary Information for

Molecular basis of the pleiotropic effects by the antibiotic amikacin on the ribosome

Savannah M. Seely[1,6], Narayan P. Parajuli[2,6], Arindam De Tarafder[2], Xueliang Ge[2], Suparna Sanyal[2,*] and Matthieu G. Gagnon[1,3,4,5,*]

[1] Department of Biochemistry and Molecular Biology, University of Texas Medical Branch, Galveston, Texas 77555, USA.

[2] Department of Cell and Molecular Biology, Biomedical Center, Uppsala University, SE-75124 Uppsala, Sweden.

[3] Department of Microbiology and Immunology, University of Texas Medical Branch, Galveston, Texas 77555, USA.

[4] Sealy Center for Structural Biology and Molecular Biophysics, University of Texas Medical Branch, Galveston, Texas 77555, USA.

[5] Institute for Human Infections and Immunity, University of Texas Medical Branch, Galveston, Texas 77555, USA.

[6] These authors contributed equally: Savannah M. Seely, Narayan P. Parajuli

* e-mail: suparna.sanyal@icm.uu.se; magagnon@utmb.edu

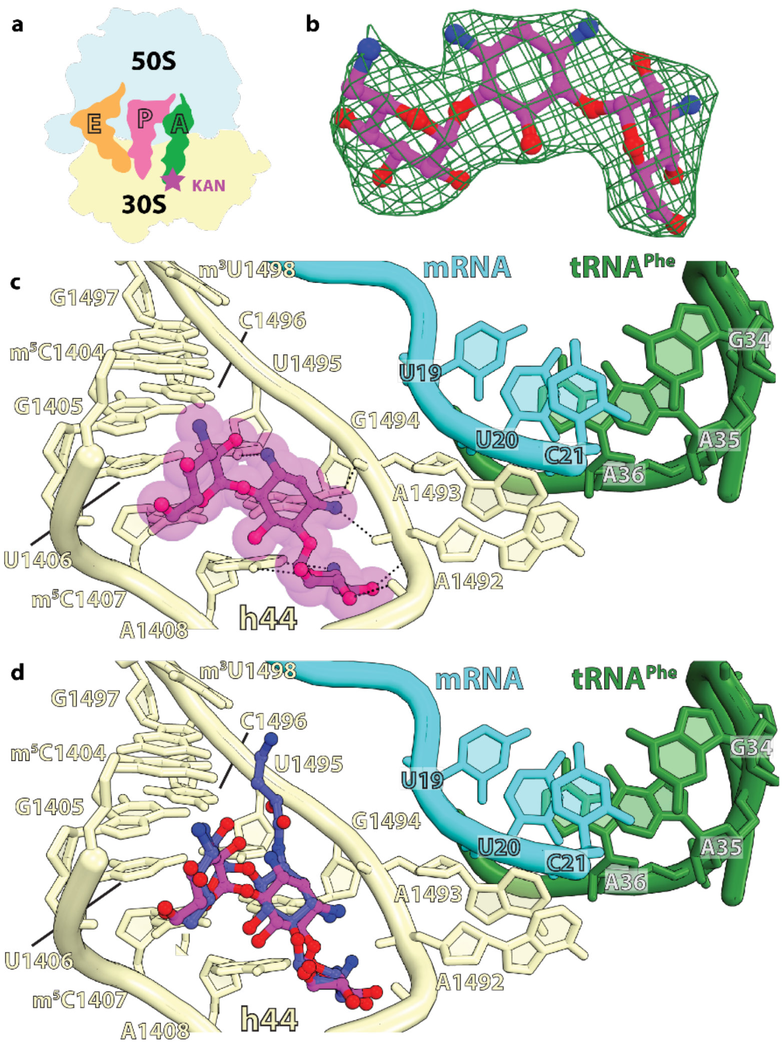

Supplementary Fig. 1. Kanamycin binds near the decoding center. a Simplified representation of the 70S ribosome with the kanamycin binding site indicated with the magenta star. **b** The unbiased ($F_o - F_c$) difference electron density map of kanamycin bound near the decoding center is contoured at 2.3σ. **c** KAN (magenta) binds within helix h44 (tan) near the decoding center. **d** Superposition of KAN (magenta) and AMK (blue) near the decoding center showing similar positioning of rings I, II and III, and unique interactions formed by the AHB moiety.

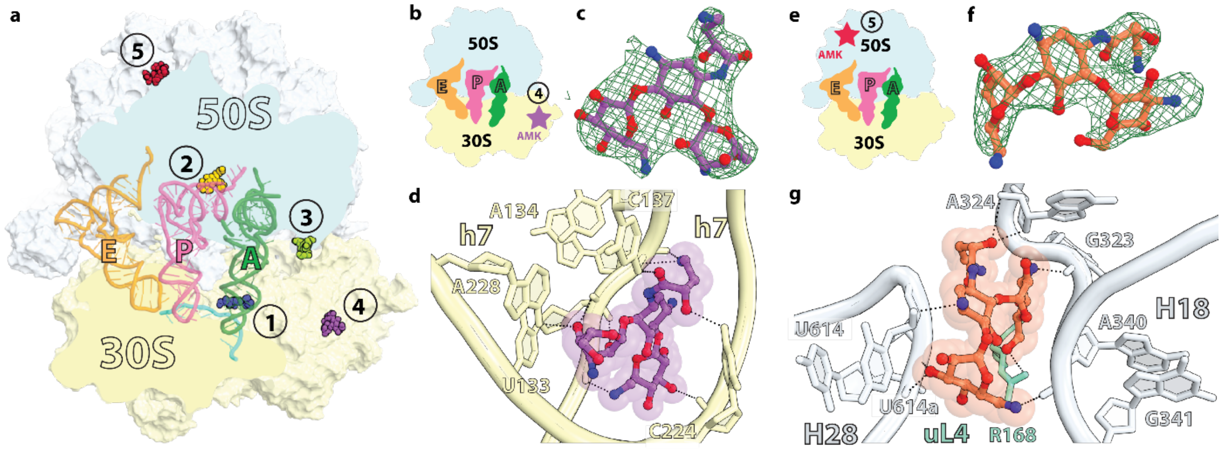

Supplementary Fig. 2. Amikacin binds in two additional sites of the *T. thermophilus* 70S ribosome. a Overview of the 70S ribosome with AMK bound in five sites. Sites 1 to 3 are described in the main text and figures. Sites 3, 4, and 5 are only observed in the crystal structure, which was obtained using a high-concentration of AMK (see Methods). In the cryo-EM structure of the *E. coli* 70S-AMK complex, only sites 1 and 2 are observed suggesting that AMK binds to sites 3, 4, and 5 with a low-affinity. **b** Simplified representation of the 70S ribosome with the amikacin binding site 4 indicated with the purple star. **c** The unbiased ($F_o - F_c$) difference electron density map of amikacin bound to site 4 is contoured at 2.3σ. **d** AMK #4 (purple) binds to the body domain of the 30S subunit interacting with 16S rRNA helix h7 (yellow). **e** Simplified representation of the 70S ribosome with the amikacin binding site 5 indicated with the orange star. **f** The unbiased ($F_o - F_c$) difference electron density map of amikacin bound to site 5 is contoured at 2.3σ. **g** AMK #5 (orange) binds in domain II of the 23S rRNA (white) interacting with helices H28 and H18 (white), and ribosomal protein uL4 (green). Note that in the *E. coli* ribosome helix H28 is shorter, which ablates this binding site.

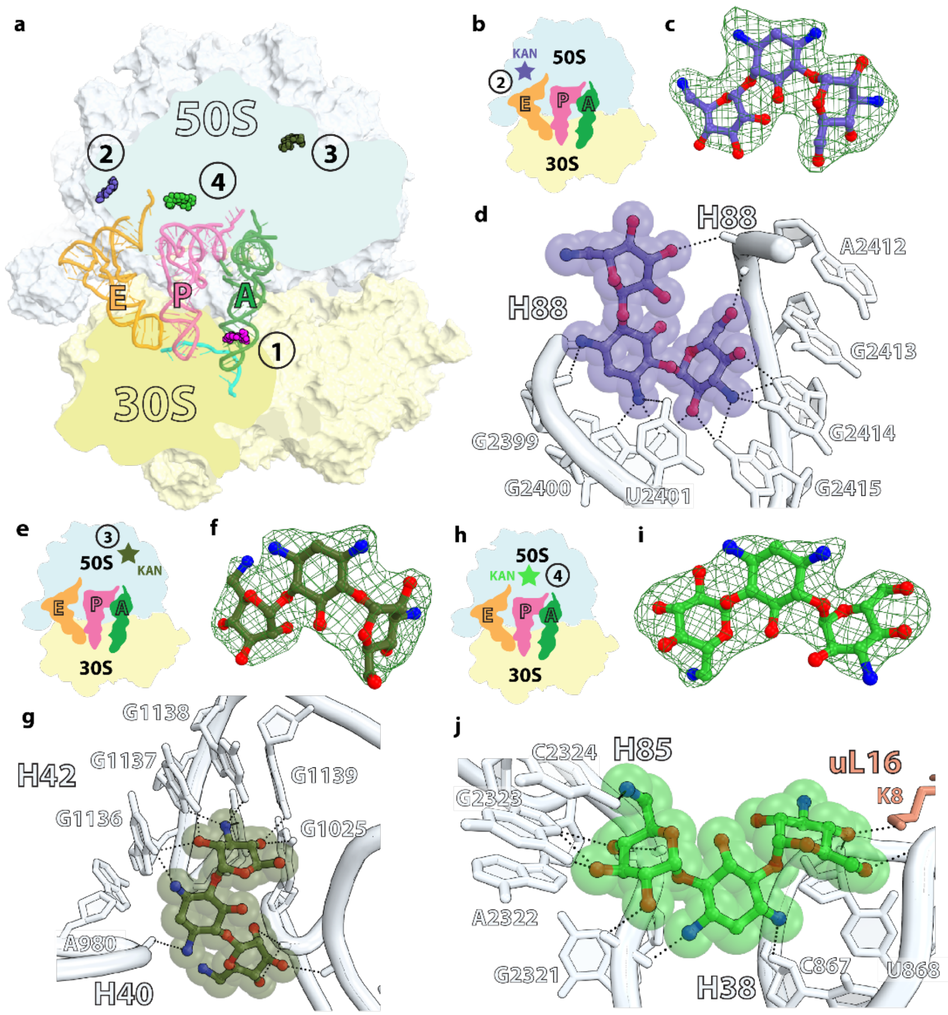

Supplementary Fig. 3. Kanamycin binds in three additional sites of the *T. thermophilus* 70S ribosome. a Overview of the 70S ribosome with KAN bound in four sites. Site 1 is described in the main text and Supplementary Fig. 1. Sites 2, 3, and 4 represent the three additional sites. **b** Simplified representation of the 70S ribosome with the kanamycin binding site 2 indicated with the purple star. **c** The unbiased ($F_o - F_c$) difference electron density map of kanamycin bound to site 2 is contoured at 2.3σ. **d** KAN at site 2 (purple) binds in domain V of the 23S rRNA (white) and interacts with helix H88. **e** Simplified representation of the 70S ribosome with the kanamycin binding site 3 indicated with the dark green star. **f** The unbiased ($F_o - F_c$) difference electron density map of kanamycin bound to site 3 is contoured at 2.3σ. **g** KAN at site 3 (dark green) binds in domain II of the 23S rRNA (white) and interacts with helices H40 and H42. **h** Simplified representation of the 70S ribosome with the kanamycin binding site 4 indicated with the green star. **i** The unbiased ($F_o - F_c$) difference electron density map of kanamycin bound to site 4 is contoured at 2.3σ. **j** KAN at site 4 (green) binds at the base of the 50S A-site finger helix H38, interacting with the latter, helix H85, and ribosomal protein uL16 (salmon).

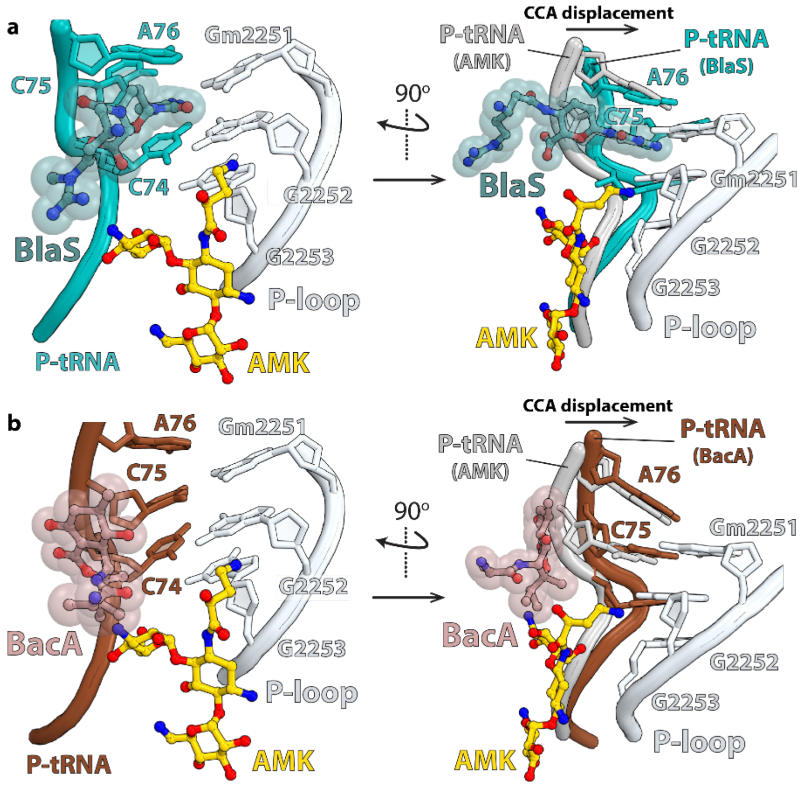

Supplementary Fig. 4. Relative binding sites of amikacin, blasticidin S (BlaS), and bactobolin A (BacA) in the large subunit P site. a Superposition of the *T. thermophilus* 70S-AMK (this work) and 70S-BlaS (PDB: 4V9Q)[1] complexes using the 23S rRNA shows the location of BlaS (teal) relative to that of AMK (yellow). The P-site tRNA taken from the 70S-BlaS complex is turquoise. **b** Similar superposition with the 70S-BacA (PDB: 4WT8)[2] complex shows the relative binding sites of AMK (yellow) and BacA (tan). The P-site tRNA taken from the 70S-BacA complex is brown. In the panels to the right, the CCA-end of the P-site tRNA from the *T. thermophilus* 70S-AMK complex (this work) is shown in gray.

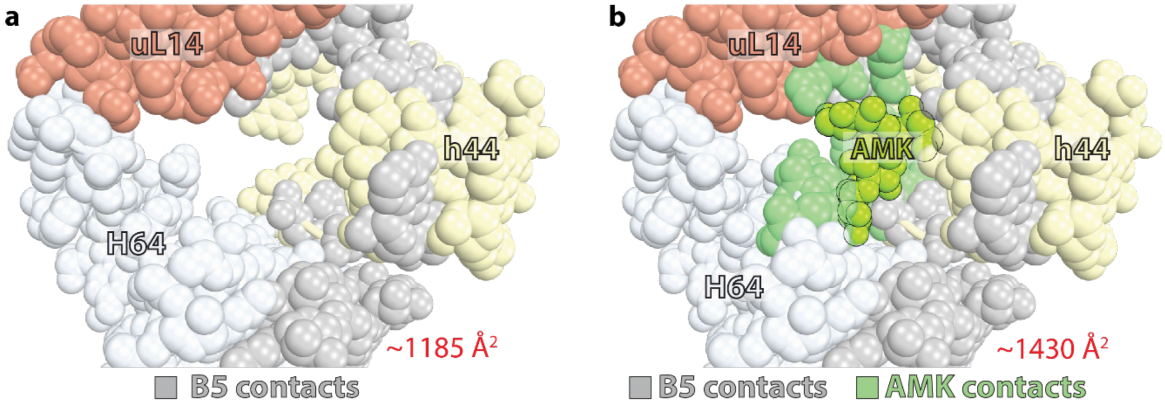

Supplementary Fig. 5. Binding of amikacin between the subunits in the *T. thermophilus* ribosome increases the contact area of inter-subunit bridge B5. a Space filling representation of inter-subunit bridge B5 components; 23S helix H64 (white), large subunit protein uL14 (salmon), and 16S helix h44 (tan) with bridge contacts displayed in gray. **b** Same as in panel (**a**) but with bound amikacin (lime) at bridge B5. Additional contacts between AMK and elements of the ribosome are shown in green.

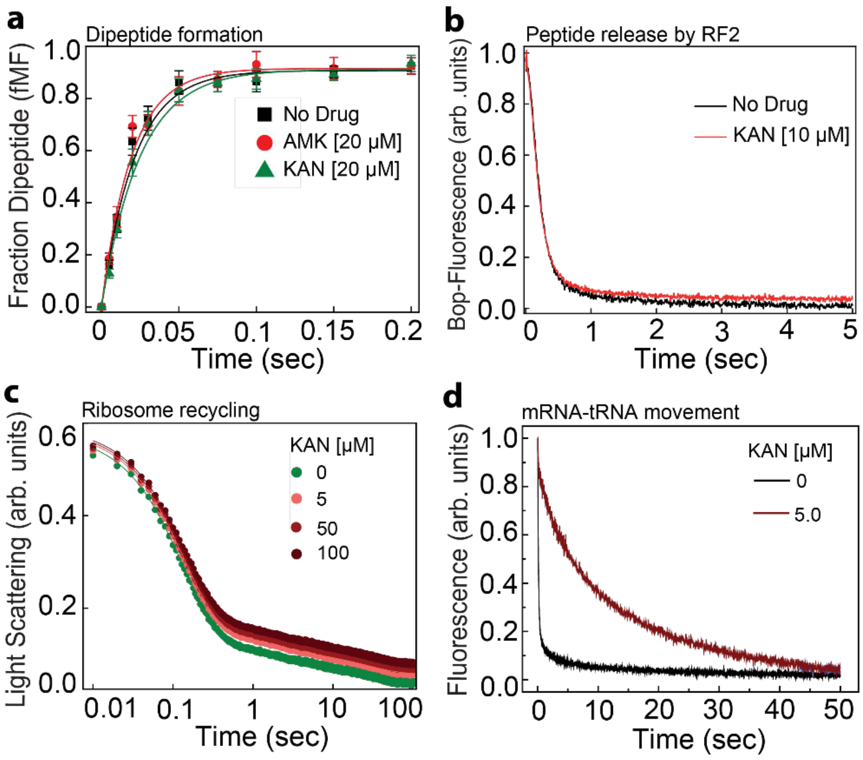

Supplementary Fig. 6. Effects of amikacin and kanamycin on the kinetics of dipeptide formation, peptide release, and ribosome recycling. a Time courses of f[^{3}H]Met-Phe dipeptide formation upon rapid mixing of EF-Tu ternary complex (TC) (5 µM) with mRNA-programmed *E. coli* 70S ribosomes (0.5 µM) carrying f[^{3}H]Met-tRNA^{fMet} in the P site, in the absence (black) and presence of AMK (red) and KAN (green) at indicated concentrations. Solid lines represent the single-exponential fit of the data. Our data show that the rates of dipeptide formation are highly similar in all three conditions. **b** The time courses of BOP-Met-Phe-Leu tripeptide release from pre-termination ribosome complexes (pre-TCs) in the absence (black) and presence of 10 µM KAN (red). The data was fitted to double exponential function and rates were estimated from predominant fast phase, which were alike in both cases. **c** Time traces of Rayleigh light scattering upon splitting of post-termination 70S ribosomes (post-TCs) into subunits by RRF and EF-G in the absence and presence of various amounts of KAN. Traces were fitted with a double exponential function. All experiments were conducted in triplicates and the figures present average data with SEM (where applicable). **d** Time traces of pyrene-mRNA+10 translocation without and with KAN (5 µM). The traces are fitted with single exponential function to obtain the rates.

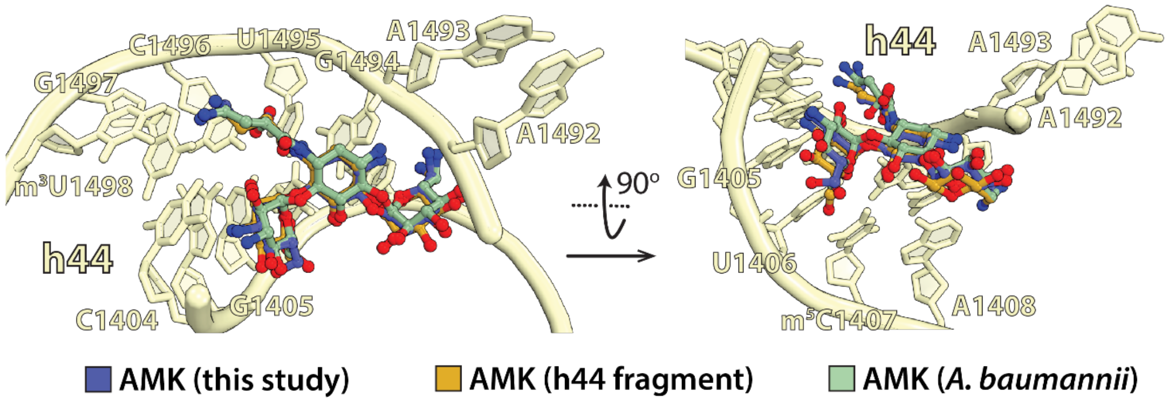

Supplementary Fig. 7. Conformation of amikacin near the decoding center. Comparison of the conformation of amikacin bound near the decoding center from the current study (blue) with that obtained using the 16S rRNA helix h44 model fragment (PDB: 4P20; gold)[3] and the *A. baumannii* 70S ribosome lacking tRNAs (PDB: 6YPU; green)[4].

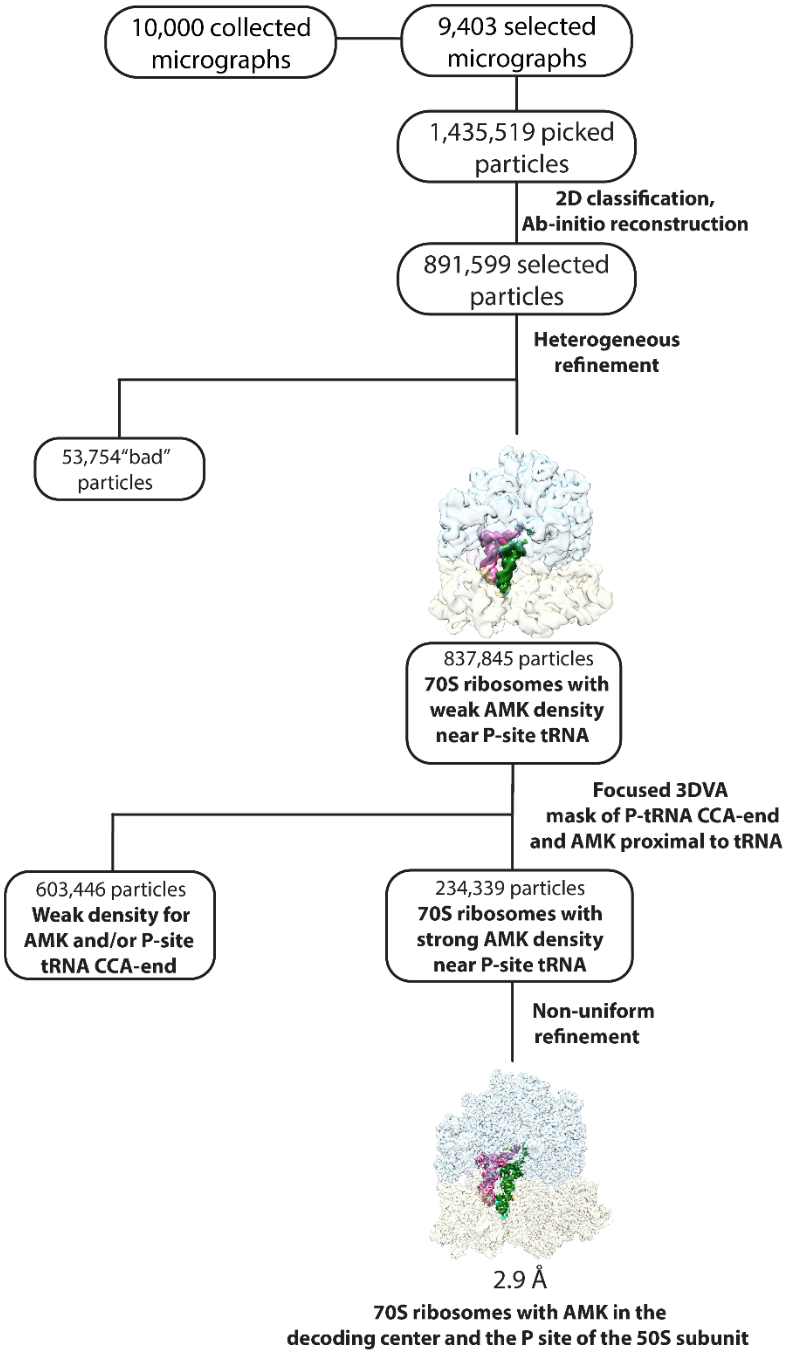

Supplementary Fig. 8. Cryo-EM data processing and particle classification workflow. All data processing steps were performed in cryoSPARC 4.1.2[5]. 10,000 micrographs were collected, from which 9,403 were selected for further processing. Following two rounds of reference-free 2D classification, the selected particles were used to generate the ab-initio volumes. ‘Heterogeneous refinement’ was performed to sort particles from the *ab-initio* 3D volumes into two groups, allowing to discard 53,754 particles, resulting in a major class average containing A-, P- and E-site tRNAs (837,845 particles). Variability analysis (3DVA) focused around the CCA-end of the

P-site tRNA and the AMK binding site in the 50S subunit proximal the tRNA was utilized to separate particles containing solid density for both the P-site tRNA and AMK near the peptidyl transferase center (234,339 particles). This process discarded 603,446 particles with weak density for AMK and/or the P-site tRNA. Non-uniform and CTF refinement yielded a reconstruction of the *E. coli* 70S ribosome with A-site Phe-$tRNA^{Phe}$, P-site $tRNA_i^{fMet}$, E-site $tRNA^{Phe}$, and AMK at nominal resolution of 2.9 Å.

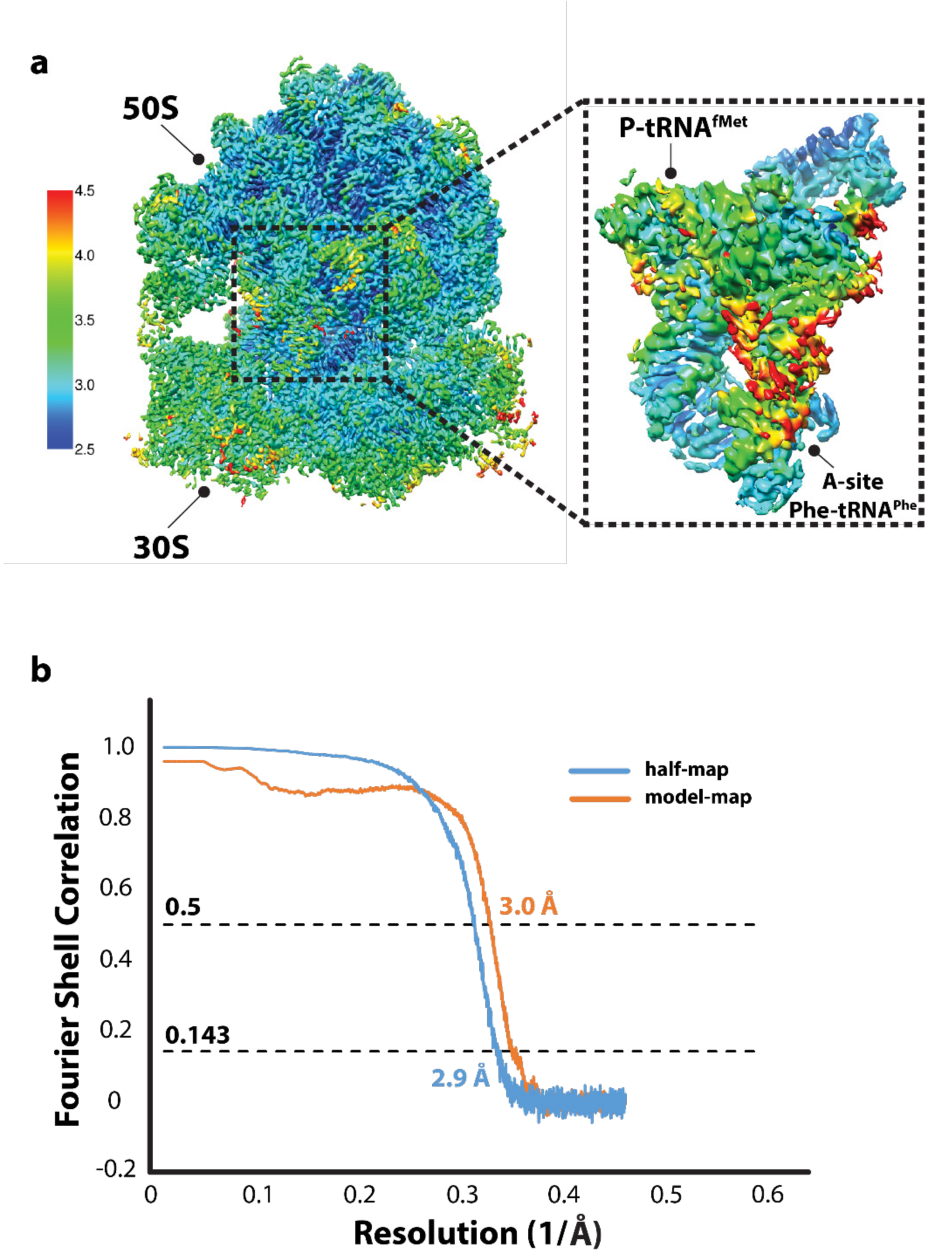

Supplementary Fig. 9. Local resolution estimation and Fourier Shell Correlation (FSC) validation. a Local resolution heat map of the *E. coli* 70S ribosome with A-site Phe-tRNA^{Phe}, P-site tRNA$_i^{fMet}$, E-site tRNA^{Phe}, and AMK shown in the range of 2.5 – 4.5 Å resolution, calculated with cryoSPARC 4.1.2 implementation of BlocRes[6]. **b** Gold-standard Fourier Shell Correlation (FSC) curves of half-maps using a 'soft mask' excluding solvent (blue) and model-map are plotted across resolution. Validation of the maps was performed in PHENIX 1.19.2[7].

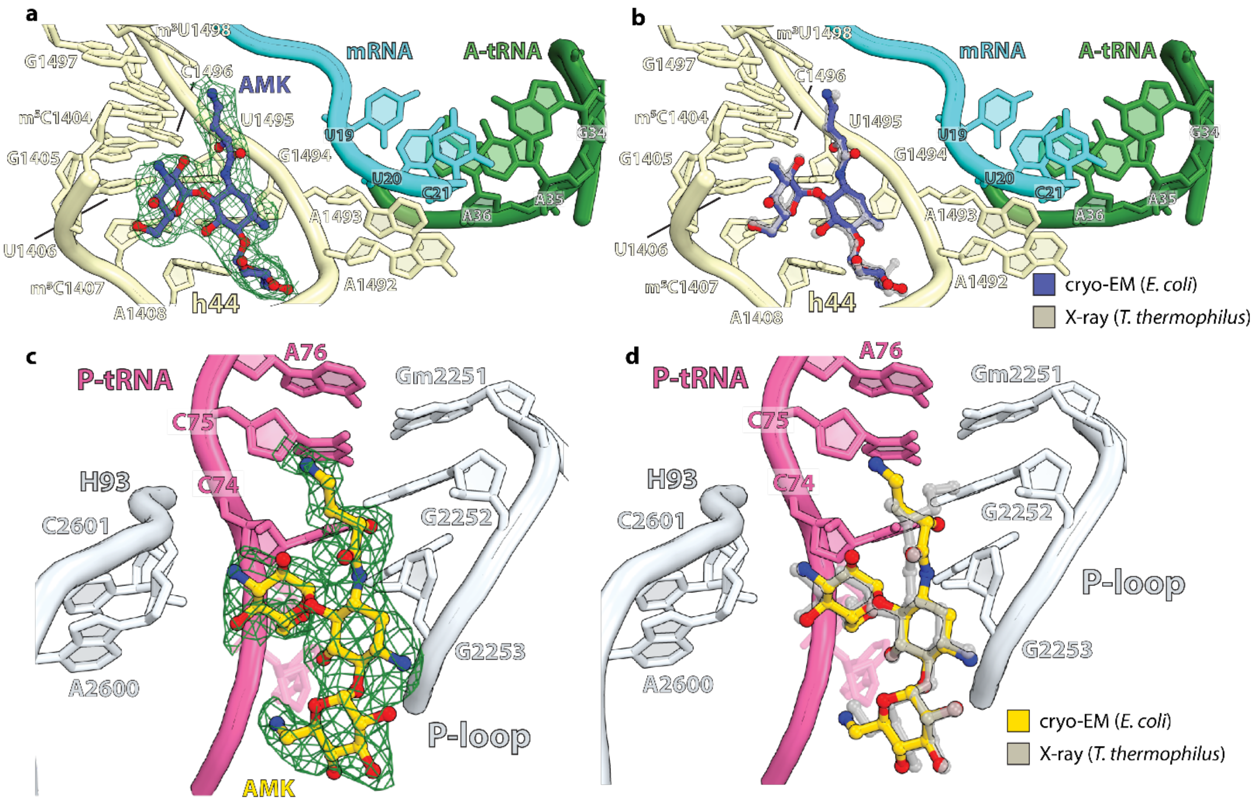

Supplementary Fig. 10. Amikacin near the decoding center and in the 50S subunit. a Cryo-EM map of amikacin bound to helix h44 near the decoding center in the *E. coli* 70S ribosome. **b** Comparison of the conformation of amikacin bound near the decoding center from the cryo-EM (blue) and crystal (gray) structures. **c** EM map of amikacin bound in the 50S subunit near the CCA-end of the P-site tRNA. **d** Comparison of the conformation of amikacin bound proximal to the peptidyl transferase center in the 50S subunit from the cryo-EM (yellow) and crystal (gray) structures.

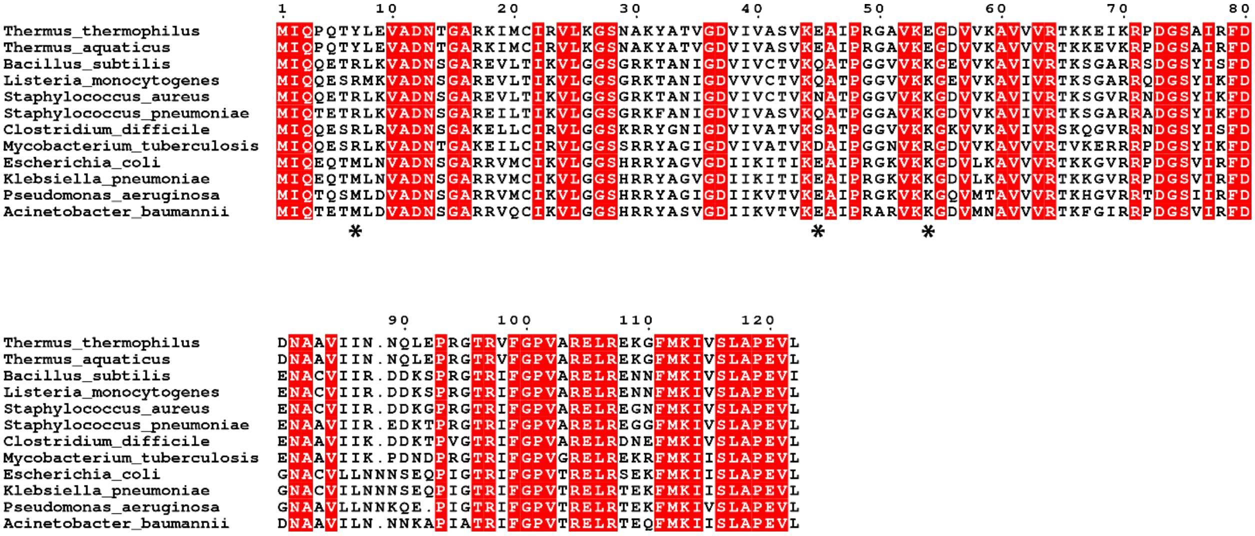

Supplementary Fig. 11. Sequence alignment of ribosomal protein uL14. The black stars denote residues 7, 45, and 54 that interact with AMK at inter-subunit bridge B5 in the *T. thermophilus* 70S ribosome. In most bacteria, a basic residue at position 54 would form a non-favorable interaction with the amine group of ring III of AMK, which may interfere with AMK binding at the ribosomal interface. The sequence alignment was generated with ClustalW 2.1[8] and the figure was made with the ESPript 3.0 server[9].

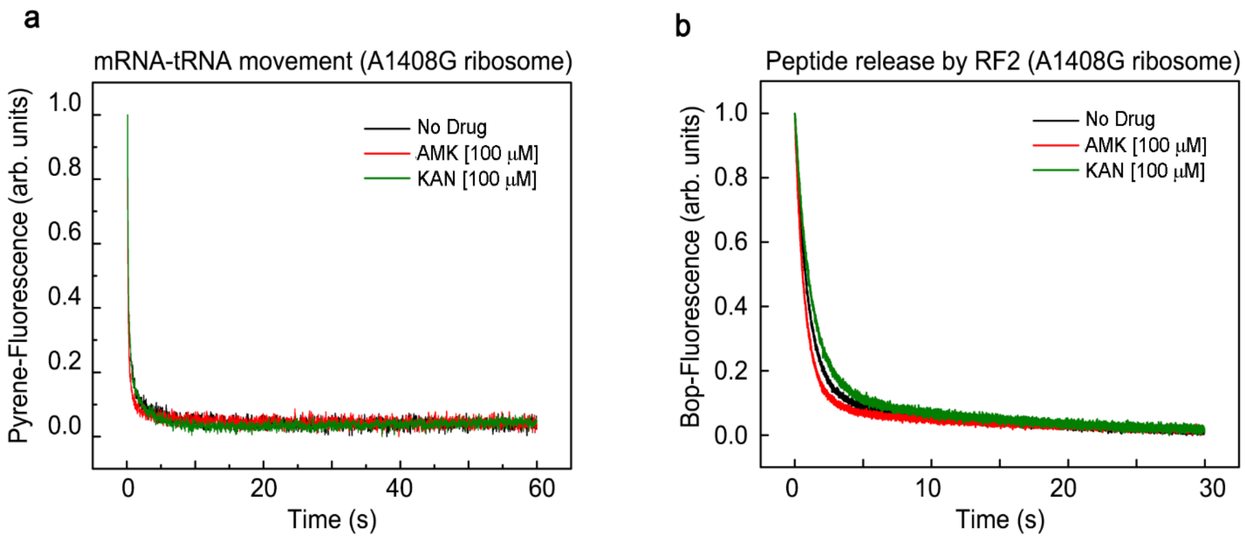

Supplementary Fig. 12. Kinetics of mRNA-tRNA translocation and RF2-mediated peptide release on the A1408G ribosomes. a Time traces obtained for the EF-G (5 μM) catalyzed movement of pyrene-labeled mRNA on the A1408G ribosomes (0.5 μM) in the absence (black) and presence of 100 μM AMK (red) and 100 μM KAN (green). The traces were fitted with single exponential function to obtain the rates. **b** RF-mediated peptide release from the A1408G ribosomes. The time courses of BOP-Met release from pre-termination ribosome complexes (0.1 μM) upon mixing with RF2 (10 μM) in the absence (black) and presence of 100 μM AMK (red) and 100 μM KAN (green). The data was fitted to double exponential function and rates (~ 1.0 s^{-1}) were estimated from predominant fast phase. The results show that the A1408G ribosomes are insensitive to AMK and KAN up to 100 μM in translation translocation and termination.

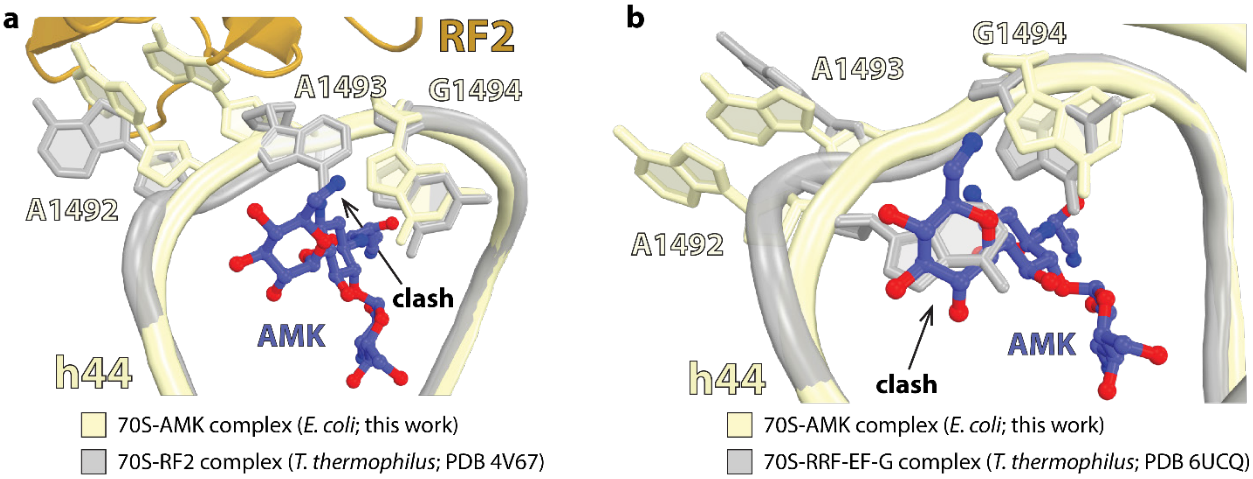

Supplementary Fig. 13. Structural basis for the interference of amikacin with RF2-mediated peptide release and ribosome recycling. a Amikacin located in h44 near the decoding center is not compatible with the conformation of A1493 upon binding of RF2 to the ribosome (PDB: 4V67)[10]. **b** Similarly, in the 70S ribosome bound to EF-G and RRF (PDB: 6UCQ)[11], A1492 would collide with amikacin.

Supplementary Table 1. Minimum inhibitory concentrations (MICs) of AMK and KAN for the *E. coli* stain SQ171 harboring wild-type and A1408G mutant ribosomes.

Antibiotic	WT *E coli* SQ171 MIC (µg/mL)			A1408G *E coli* SQ171 MIC (µg/mL)		
	Exp1	Exp2	Exp3	Exp1	Exp2	Exp3
**KAN**	8	8	8	>256	256	256
**AMK**	1	1	1	32	16	16

Supplementary Table 2. X-ray data collection, refinement and validation statistics

	***T. thermoplilus* 70S-AMK (PDB 8EV6)**	***T. thermoplilus* 70S-KAN (PDB 8EV7)**
**Data collection**		
Space group	$P2_12_12_1$	$P2_12_12_1$
Cell dimensions		
a, *b*, *c* (Å)	210.1, 446.8, 620.2	209.4, 447.6, 617.8
α, β, γ (°)	90, 90, 90	90, 90, 90
Resolution (Å)	187.65 – 2.95 (3.12 – 2.95)[a]	187.07 – 2.89 (3.06 – 2.89)[b]
R_{sym} (%)	28.5 (237.8)	26.5 (207.5)
$I/\sigma(I)$	5.59 (0.67)	6.81 (0.80)
$CC_{1/2}$	99.4 (11.3)	99.5 (18.7)
Completeness (%)	99.0 (97.7)	98.7 (94.8)
Redundancy	4.7 (4.7)	4.6 (4.7)
**Refinement**		
Resolution (Å)	2.95	2.89
No. reflections	1,198,299	1,260,633
R_{work} / R_{free}	22.3 / 27.2	21.6 / 26.3
No. of non-hydrogen atoms		
Protein	90,976	90,976
RNA	200,224	200,225
Ions (Mg/Zn/Fe)	2,333/12/2	2,259/12/2
Waters	2,328	2,125
Amikacin/Kanamycin	8	7
Average *B* factors (Å^2)		
Protein	75.9	70.0
RNA	73.3	67.4
Ions	62.4	59.7
Waters	58.0	55.7
MolProbity score	2.86	2.77
Clashscore	10.66	9.46
R.m.s. deviations		
Bond lengths (Å)	0.004	0.004
Bond angles (°)	0.889	0.835
Ramachandran plot		
Favored (%)	89.91	91.24
Allowed (%)	8.71	7.43
Disallowed (%)	1.38	1.33

Diffraction data from single crystals were used to determine the structures. Values in parentheses are for the highest-resolution shell.

[a] $I/\sigma(I) = 2$ at 3.34 Å resolution.

[b] $I/\sigma(I) = 2$ at 3.20 Å resolution.

Supplementary Table 3. Cryo-EM data collection, refinement and validation statistics.

	***E. coli* 70S-AMK (EMD-40882) (PDB 8SYL)**
**Data collection and processing**	
Magnification	105,000x
Voltage (kV)	300
Electron exposure (e^{-}/Å^2)	40.6
Defocus range (µm)	–0.7 to –2.0
Detector	Gatan K3
Pixel size (Å)	0.839
Symmetry imposed	C1
Initial particle images (no.)	891,599
Final particle images (no.)	234,339
Map resolution (Å)	2.9
FSC threshold	0.143
**Refinement**	
Initial model used (PDB code)	8EKC
Model resolution (Å)	3.0
FSC threshold	0.5
Map sharpening *B* factor (Å^2)	–40
CC_{mask}	0.88
MolProbity score	1.74
Clashscore	8.34
Model composition	
Chains	57
Non-hydrogen atoms	144,592
Protein residues	5,549
RNA residues	4,728
Ions (Mg / Zn)	474 / 2
Waters	74
Amikacin	2
Average *B* factors (Å^2)	
Protein	68.9
RNA	71.8
Ions	45.7
Waters	40.2
R.m.s. deviations	
Bond lengths (Å)	0.004
Bond angles (°)	0.551
Ramachandran plot	
Favored (%)	95.88
Allowed (%)	3.97
Disallowed (%)	0.15

Supplementary References

1. Svidritskiy, E., Ling, C., Ermolenko, D.N. & Korostelev, A.A. Blasticidin S inhibits translation by trapping deformed tRNA on the ribosome. *Proc. Natl. Acad. Sci. USA* **110**, 12283-8 (2013).
2. Amunts, A. et al. Bactobolin A binds to a site on the 70S ribosome distinct from previously seen antibiotics. *J. Mol. Biol.* **427**, 753-755 (2015).
3. Kondo, J., Francois, B., Russell, R.J., Murray, J.B. & Westhof, E. Crystal structure of the bacterial ribosomal decoding site complexed with amikacin containing the gamma-amino-alpha-hydroxybutyryl (haba) group. *Biochimie* **88**, 1027-31 (2006).
4. Nicholson, D., Edwards, T.A., O'Neill, A.J. & Ranson, N.A. Structure of the 70S Ribosome from the Human Pathogen Acinetobacter baumannii in Complex with Clinically Relevant Antibiotics. *Structure* **28**, 1087-1100 e3 (2020).
5. Punjani, A., Rubinstein, J.L., Fleet, D.J. & Brubaker, M.A. cryoSPARC: algorithms for rapid unsupervised cryo-EM structure determination. *Nat. Methods* **14**, 290-296 (2017).
6. Cardone, G., Heymann, J.B. & Steven, A.C. One number does not fit all: mapping local variations in resolution in cryo-EM reconstructions. *J. Struct. Biol.* **184**, 226-36 (2013).
7. Afonine, P.V. et al. New tools for the analysis and validation of cryo-EM maps and atomic models. *Acta Crystallogr. D Struct. Biol.* **74**, 814-840 (2018).
8. Larkin, M.A. et al. Clustal W and Clustal X version 2.0. *Bioinformatics* **23**, 2947-8 (2007).
9. Robert, X. & Gouet, P. Deciphering key features in protein structures with the new ENDscript server. *Nucl. Acids Res.* **42**, W320-4 (2014).
10. Korostelev, A. et al. Crystal structure of a translation termination complex formed with release factor RF2. *Proc. Natl. Acad. Sci. USA* **105**, 19684-9 (2008).
11. Zhou, D., Tanzawa, T., Lin, J. & Gagnon, M.G. Structural basis for ribosome recycling by RRF and tRNA. *Nat. Struct. Mol. Biol.* **27**, 25-32 (2020).
