# Supplementary material for: Molecular basis of the pleiotropic effects by the antibiotic amikacin on the ribosome
Source: Nat Commun. 2023 Aug 3;14:4666. doi: 10.1038/s41467-023-40416-5 (PMC10400623; doi:10.1038/s41467-023-40416-5)
Supplement: Supplementary file 3 — Reporting Summary [file 41467_2023_40416_MOESM3_ESM.pdf]

nature portfolio

Corresponding author(s): Matthieu G. Gagnon
Suparna Sanyal

Last updated by author(s): Jul 19, 2023

Reporting Summary

Nature Portfolio wishes to improve the reproducibility of the work that we publish. This form provides structure for consistency and transparency in reporting. For further information on Nature Portfolio policies, see our Editorial Policies and the Editorial Policy Checklist.

Statistics

For all statistical analyses, confirm that the following items are present in the figure legend, table legend, main text, or Methods section.

n/a	Confirmed	
☐	☒	The exact sample size (*n*) for each experimental group/condition, given as a discrete number and unit of measurement
☐	☒	A statement on whether measurements were taken from distinct samples or whether the same sample was measured repeatedly
☒	☐	The statistical test(s) used AND whether they are one- or two-sided<br>*Only common tests should be described solely by name; describe more complex techniques in the Methods section.*
☒	☐	A description of all covariates tested
☒	☐	A description of any assumptions or corrections, such as tests of normality and adjustment for multiple comparisons
☐	☒	A full description of the statistical parameters including central tendency (e.g. means) or other basic estimates (e.g. regression coefficient) AND variation (e.g. standard deviation) or associated estimates of uncertainty (e.g. confidence intervals)
☒	☐	For null hypothesis testing, the test statistic (e.g. *F*, *t*, *r*) with confidence intervals, effect sizes, degrees of freedom and *P* value noted<br>*Give P values as exact values whenever suitable.*
☒	☐	For Bayesian analysis, information on the choice of priors and Markov chain Monte Carlo settings
☒	☐	For hierarchical and complex designs, identification of the appropriate level for tests and full reporting of outcomes
☒	☐	Estimates of effect sizes (e.g. Cohen's *d*, Pearson's *r*), indicating how they were calculated

Our web collection on statistics for biologists contains articles on many of the points above.

Software and code

Policy information about availability of computer code

Data collection	X-ray diffraction data was collected at beamlines 24ID-C and 24ID-E at the Advanced Photon Source (Argonne National Laboratory) using NE-CAT remote access software (v. 6.2.0). The cryo-EM data was collected with SerialEM (v. 4-0-19).
Data analysis	X-ray crystallographic data were integrated and scaled using the XDS software (June 17, 2015). The structures were built in COOT (v. 0.8.9.1) and refined using the PHENIX software (v. 1.14). All figures showing atomic models were generated using the PyMol software (v. 2.1.0). The cryo-EM data was analyzed with CryoSPARC (v. 4.1.2), Phenix and Molprobity (v. 1.19.2-4158), Coot 0.9.8.7, PyMOL (v. 2.1.0), Chimera (v. 1.14). The molecular models of amikacin and kanamycin were generated using the ChemDraw Professional (v. 16) software, and the kinetics data analyzed with the OriginPro (v. 2016).

For manuscripts utilizing custom algorithms or software that are central to the research but not yet described in published literature, software must be made available to editors and reviewers. We strongly encourage code deposition in a community repository (e.g. GitHub). See the Nature Portfolio guidelines for submitting code & software for further information.

Data

Policy information about availability of data

All manuscripts must include a data availability statement. This statement should provide the following information, where applicable:

- Accession codes, unique identifiers, or web links for publicly available datasets
- A description of any restrictions on data availability
- For clinical datasets or third party data, please ensure that the statement adheres to our policy

Coordinates and structure factors of the Thermus thermophilus 70S ribosome complexes have been deposited in the Protein Data Bank under the accession codes:
- 8EV6 for the T. thermophilus 70S ribosome in complex with amikacin [https://doi.org/10.2210/pdb8EV6/pdb]
- 8EV7 for the T. thermophilus 70S ribosome in complex with kanamycin [https://doi.org/10.2210/pdb8EV7/pdb]

The cryo-EM map of the Escherichia coli 70S ribosome bound to AMK has been deposited in the Electron Microscopy Data Bank (EMDB) under the accession code EMD-40882 [https://www.ebi.ac.uk/pdbe/entry/emdb/EMD-40882], and the corresponding atomic coordinates in the PDB under the accession code 8SYL [https://doi.org/ 10.2210/pdb8SYL/pdb].

Human research participants

Policy information about studies involving human research participants and Sex and Gender in Research.

Reporting on sex and gender	Not applicable
Population characteristics	Not applicable
Recruitment	Not applicable
Ethics oversight	Not applicable

Note that full information on the approval of the study protocol must also be provided in the manuscript.

Field-specific reporting

Please select the one below that is the best fit for your research. If you are not sure, read the appropriate sections before making your selection.

☒ Life sciences ☐ Behavioural & social sciences ☐ Ecological, evolutionary & environmental sciences

For a reference copy of the document with all sections, see nature.com/documents/nr-reporting-summary-flat.pdf

Life sciences study design

All studies must disclose on these points even when the disclosure is negative.

Sample size	The biochemical experiments were conducted in triplicates following the standard practice. The cryo-EM dataset contains 10,000 micrographs which, following focused 3D variability analysis, yielded to the 70S ribosome complex bound to amikacin, mRNA, and tRNAs (234,339 particles). No statistical method was used for determining sample size.
Data exclusions	During the analysis of EM images, those with thick ice or excessive particle motion were excluded. Two-dimension (2D) and three-dimension (3D) classification of the particles was performed to exclude non-specimen related particles.
Replication	Biochemical experiments were successfully conducted in triplicates. One additional cryo-EM dataset containing 100 uM amikacin was collected from an independent sample, which confirmed the findings.
Randomization	The kinetics experiments were intrinsically randomized, as the experimental groups constituted equally sized aliquots of a common homogeneous custom-made cell-free translation system. For cross-validation during crystallographic model building and refinement, an R-free set was used. For the cryo-EM data analysis, computational unsupervised particle classification employs the maximum likelihood estimation algorithm which includes randomization. Classification was repeated three times varying the number of classes, which yielded similar particle distribution among the clusters.
Blinding	Blinding is not applicable to the crystal structures generated because they are not subjective assessments of the experimenter. For the cryo-EM data analysis, particle classification was performed in CryoSPARC (v. 4.1.2) which uses a maximum likelihood estimation algorithm, blinding the user to group allocation during 3D particle classification. Classification was repeated three times varying the number of classes, yielding similar particle distribution among the clusters.

Reporting for specific materials, systems and methods

We require information from authors about some types of materials, experimental systems and methods used in many studies. Here, indicate whether each material, system or method listed is relevant to your study. If you are not sure if a list item applies to your research, read the appropriate section before selecting a response.

Materials & experimental systems

n/a	Involved in the study
☒	☐ Antibodies
☒	☐ Eukaryotic cell lines
☒	☐ Palaeontology and archaeology
☒	☐ Animals and other organisms
☒	☐ Clinical data
☒	☐ Dual use research of concern

Methods

n/a	Involved in the study
☒	☐ ChIP-seq
☒	☐ Flow cytometry
☒	☐ MRI-based neuroimaging
